# Axl is not an indispensable factor for Zika virus infection in mice

**DOI:** 10.1099/jgv.0.000886

**Published:** 2017-08-08

**Authors:** Zhao-Yang Wang, Zai Wang, Zi-Da Zhen, Kai-Hao Feng, Jing Guo, Na Gao, Dong-Ying Fan, Dai-Shu Han, Pei-Gang Wang, Jing An

**Affiliations:** ^1^​ Department of Microbiology, School of Basic Medical Sciences, Capital Medical University, Beijing 100069, PR China; ^2^​ Institute of Clinical Medicine, China-Japan Friendship Hospital, Beijing 100029, PR China; ^3^​ Institute of Basic Medical Sciences, Chinese Academy of Medical Sciences, Peking Union Medical College, Beijing 100730, PR China; ^4^​ Center of Epilepsy, Beijing Institute for Brain Disorders, Beijing 100093, PR China

**Keywords:** ZIKV, Axl, receptor, brain, *in vivo* role

## Abstract

Recently, Zika virus (ZIKV) outbreak has been associated with a sharp increase in cases of Guillain–Barré syndrome and severe fetal abnormalities. However, the mechanism underlying the interaction of ZIKV with host cells is not yet clear. Axl, a receptor tyrosine kinase, is postulated as a receptor for ZIKV entry; however, its *in vivo* role during ZIKV infection and its impact on the outcome of the disease have not been fully characterized and evaluated. Moreover, there are contradictory results on its involvement in ZIKV infection. Here we utilized Axl-deficient mice (Axl^−/−^) and their littermates (Axl^+/−^) to study the *in vivo* role of Axl in ZIKV infection. Our results showed that both Axl^+/−^ and Axl^−/−^ suckling mice supported the replication of ZIKV and presented clinical manifestations. No significant difference has been found between Axl-deficient mice and their littermates in terms of the survival rate, clinical manifestations, viral load, ZIKV distribution and histopathological changes in major organs. These results therefore indicate that Axl is not an indispensable factor for ZIKV infection in mice.

## Abbreviations

DENV, dengue virus; GFAP, glial fibrillary acidic protein; HE, hematoxylin and eosin; MEM, minimum essential medium; p.i., post-infection; ZIKV, zika virus.

## Introduction

Zika virus (ZIKV) belongs to the *Flavivirus* genus within the *Flaviviridae* family [1, 2], which includes a number of human pathogens such as dengue virus (DENV), yellow fever virus, West Nile virus and Japanese encephalitis virus and threats to global human health. Since 2007, ZIKV has infected several million people in more than 70 countries and caused thousands of microcephaly in newborns and Guillain–Barré syndrome in adults [3, 4]. Clinical and animal experimental data have shown that the nervous system is the main target for ZIKV infection [5–8]. In addition, ZIKV can be detected in urine for a long time, suggesting that kidneys may be susceptible to ZIKV infection [9]. Testis damage caused by ZIKV infection in mice has recently been reported [10–12], but its implication in humans requires a long-term investigation. In the last year, great progress was achieved to reveal the pathogenesis of ZIKV. However, the molecular mechanism underlying the entry process of ZIKV still needs to be elucidated.

Several studies have speculated that Axl is a potential entry receptor for ZIKV. Axl is a receptor tyrosine kinase and together with two other similar proteins, Tyro3 and Mer, composes the family of TAM receptors which have been shown to mediate entry of DENV [13, 14]. Axl is mainly expressed in monocytes and cells from the immune-privileged regions such as the nervous system and reproductive system [13]. Congruency of the distribution of Axl and the tissue tropism of ZIKV made researchers highlight its role as a candidate receptor for ZIKV [15], and some experimental results from different groups also supported this idea. Hamel *et al*. tested some entry and/or adhesion factors, which are crucial for flavivirus entry, and found that Axl is likely to play a major role in ZIKV infection [16]. Savidis *et al.* performed a functional genomic screen and conceived Axl as an entry factor for ZIKV infection [17]. Liu *et al.* showed that Axl could mediate productive infection of ZIKV in human endothelial cells [18]. Ma *et al.* indicated that the ZIKV infection was correlated well with the expression of Axl in testis and epididymis in mice [11]. Very recently, Meertens *et al.* used an engineered Axl decoy receptor and the Axl kinase inhibitor R428 to demonstrate Axl as a receptor for ZIKV entry into human glial cells [19]. Moreover, their results showed that ZIKV infection activated Axl kinase activity, which down-regulated IFN signalling and facilitated infection [19]. All of the above results suggested that Axl acts as a crucial factor for mediating ZIKV entry *in vitro.*

In spite of these results, there are very limited investigations regarding the *in vivo* role of Axl in ZIKV infection and the influence of Axl deficiency on the outcome of Zika disease. The impact of Axl deficiency has merely been tested on eyes [20] and the male reproductive system [10], and the contradictory results were documented regarding Axl’s involvement in testis damage caused by ZIKV [10, 11]. However, the anti-IFN receptor antibody used in these studies impaired the effects of Axl on IFN signalling and may underestimate the contribution of Axl during ZIKV infection. A strategy for a ZIKV animal model rather than IFN receptor disruption is thus needed to investigate the *in vivo* role of Axl.

In the current study, we used Axl-deficient mice (C57BL/6) and SJL mice which are susceptible to ZIKV infection [21] to generate Axl^−/−^ mice and their littermates (Axl^+/−^) with a SJL background. When intracerebrally injected with ZIKV, these mice gradually displayed clinical manifestations such as sloth, bradykinesia, which is a sign of moving slowly or with difficultly, and stopped body weight gain. We found that both Axl-deficient suckling mice and their littermates supported the replication of ZIKV and presented similar pathological changes in major relevant organs and similar survival rates. No significant difference was observed between Axl-deficient mice and their littermates. Our results therefore indicated that Axl is not an indispensable factor for ZIKV infection in mice.

## Results

**Fig. 1.**
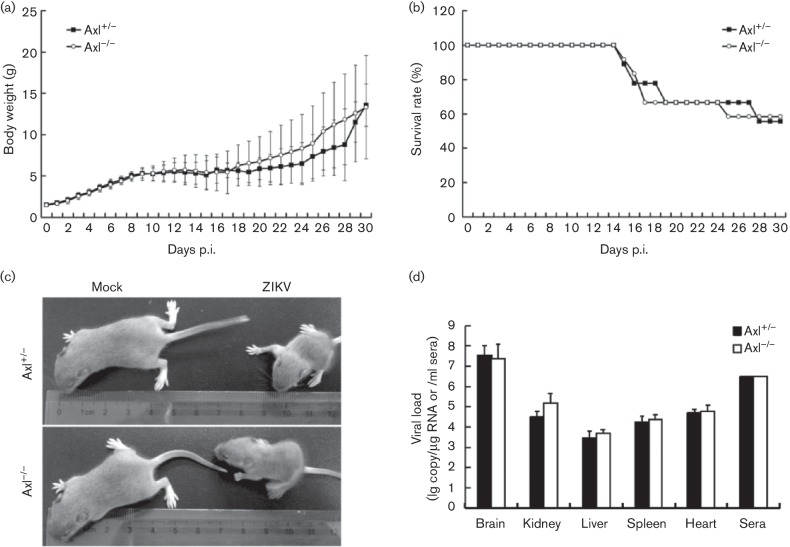
Signs of infection and the viral load in ZIKV-infected mice. (a, b) Changes of body weight (a) and survival rates (b) of suckling mice intracerebrally injected with 100 pfu ZIKV within 72 h after birth (*n*=12 for Axl^−/−^ and *n*=9 for Axl^+/−^). (c) Representative photo of mice injected with ZIKV or PBS (mock) (Axl^+/−^ and Axl^−/−^ as indicated) at 14 days p.i. (d) Viral loads in major organs and sera of infected mice at 10 days p.i. were determined by qRT-PCR (*n*=3) and expressed as the copy number per microgram of total RNA or per milliliter of sera. The copy number of the virus was standardized with ZIKV RNA transcripted *in vitro*.

To investigate whether Axl plays a crucial role in ZIKV infection in mice, newborn Axl^−/−^ and Axl^+/−^ mice generated from Axl^−/−^ x Axl^+/−^ were characterized by genotyping PCR (Fig. S1, available in the online Supplementary Material) and were injected intracerebrally with 100 pfu ZIKV (CAS-ZK01 strain) within 72 h after birth. The body weight change and survival were observed every day in the following 30 days. Our results showed that both Axl^−/−^ and their littermates (Axl^+/−^) stopped weight gain at the ninth day post infection (p.i.) and remained unchanged for about 10 days. Then the weight gained recovered at about 19 days p.i. (Fig. 1a). At 14 days p.i., mouse death occurred in both Axl^−/−^ and their littermates (Fig. 1b). The body sizes of surviving mice were significantly smaller than the mock control injected intracerebrally with PBS (Fig. 1c). By the end of the observation time, 41.6 % Axl^−/−^ mice (5/12) and 44.4 % (4/9) of their littermates died (Fig. 1b). No statistically significant difference was found between Axl^−/−^ and their littermates in terms of the body weight change and survival rate.

Viral loads in major organs including the brain, heart, kidney, liver and spleen as well as serum were measured by qRT-PCR at 10 days p.i. The highest viral load with 10^8^ copies µg^−1^ total RNA was detected in brain (Fig. 1d), similar to previous reports [5]. In all organs and serum tested, similar viral loads ranging from 10^6^–10^8^ copies µg^−1^ total RNA or ml^-1^ sera were detected in Axl^−/−^ mice and their littermates (Fig. 1d). These results indicate that ZIKV has a similar organ distribution pattern and replicates at a similar level in Axl^−/−^ mice and their littermates, which results in a congruent infection outcome.

**Fig. 2.**
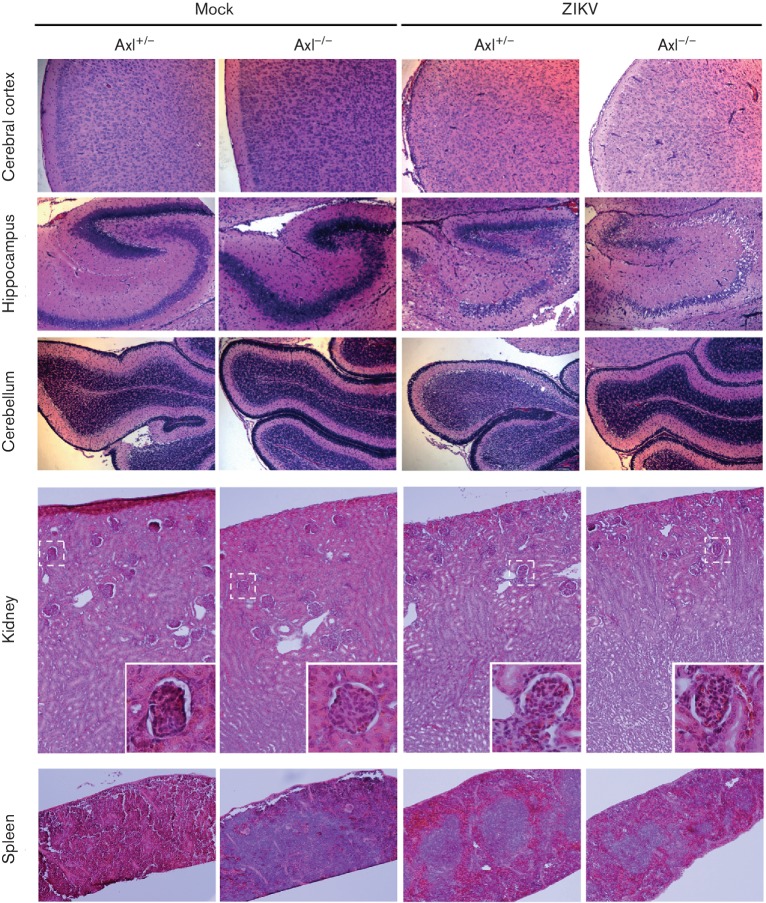
HE staining of the brain, kidney and spleen from ZIKV-infected mice. Axl-deficient (Axl^−/−^) newborn mice and their littermates (Axl^+/−^) were intracerebrally injected with 100 pfu ZIKV or the same volume of PBS (mock). The brain, kidney and spleen were harvested at 10 days p.i. and processed for HE staining as indicated. Magnification: ×40 for the cerebral cortex, hippocampus and cerebellum; ×100 for the kidney and spleen; ×400 for insets in the pictures of the kidney.

To analyse the pathological changes caused by ZIKV infection, organs that might be damaged by ZIKV infection, including the brain, heart, kidney, liver and spleen, were dissected at 10 days p.i., and sections were subjected to hematoxylin and eosin (HE) staining. The testis was not fully developed at the time, thus it was not analysed in our study. In contrast to mice injected intracerebrally with PBS, mice injected with ZIKV showed prominent pathological changes in both Axl^−/−^ and their littermates. Among all organs observed, there were obvious pathological changes in the brain including hyperemia, neuron death, neural cell disorganization and infiltration of inflammatory cells in the hippocampus and the cerebral cortex (Fig. 2). There was remarkable atrophy of the glomerulus, and the renal cortex became thin and demonstrated infiltration of inflammatory cells and congestion. Moreover, enlarged white pulps and the appearance of germinal centres were observed in the spleen (Fig. 2). The heart and liver showed no other conspicuous changes except for infiltration of inflammatory cells (Fig. S2) in the liver. All these pathological changes were observed in both Axl^−/−^ mice and their littermates to the same extent, indicating that Axl expression did not have an impact on the pathological changes induced by ZIKV infection.

**Fig. 3.**
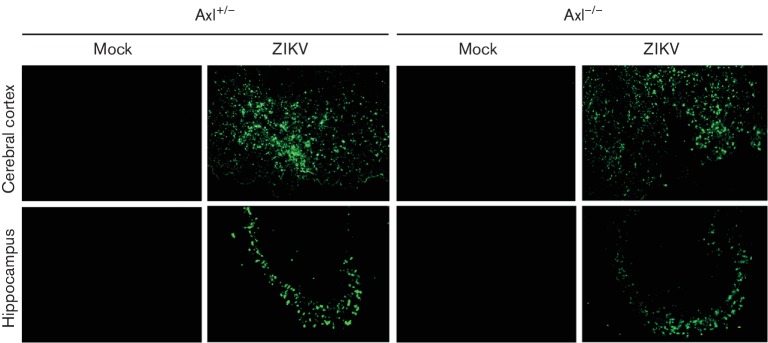
Immunofluorescent staining of ZIKV antigens in the cerebral cortex and hippocampus from ZIKV-infected mice. Axl-deficient (Axl^−/−^) newborn mice and their littermates (Axl^+/−^) were intracerebrally injected with 100 pfu ZIKV and the brains were harvested at 10 days p.i. and processed for immunofluorescene of ZIKV antigens (green). Uninfected mice were used as mock control. Magnification: ×100.

To determine the distribution of ZIKV in different organs, cyrosections of these organs were subjected to immunofluorescence staining. In the brains of Axl^−/−^ mice and their littermates, ZIKV antigens were mainly distributed in the cerebral cortex and hippocampus (Fig. 3). Cryosections of the major organs were further subjected to immunohistochemical staining. ZIKV antigens were detected in the brain with the highest signal intensity (Fig. S3), consistent with the highest level of viral load (Fig. 1d) and obvious histopathological changes. Additionally positive immunoreactivity of ZIKV antigens was also observed in the kidney and spleen (Fig. S3), also consistent with high viral loads and histopathological changes. The heart and liver also showed some positive signal of ZIKV antigens although there were no obvious histopathological changes in the heart. However, some unspecific positive immunoreactivity was observed in the spleen of mock-treated mice, which may be due to non-specific binding of an antibody with an Fc receptor on the surface of immune cells such as B lymphocytes and macrophages, and these cells richly distribute in this organ. Importantly, no difference was found between the Axl^−/−^ mice and their littermates.

**Fig. 4.**
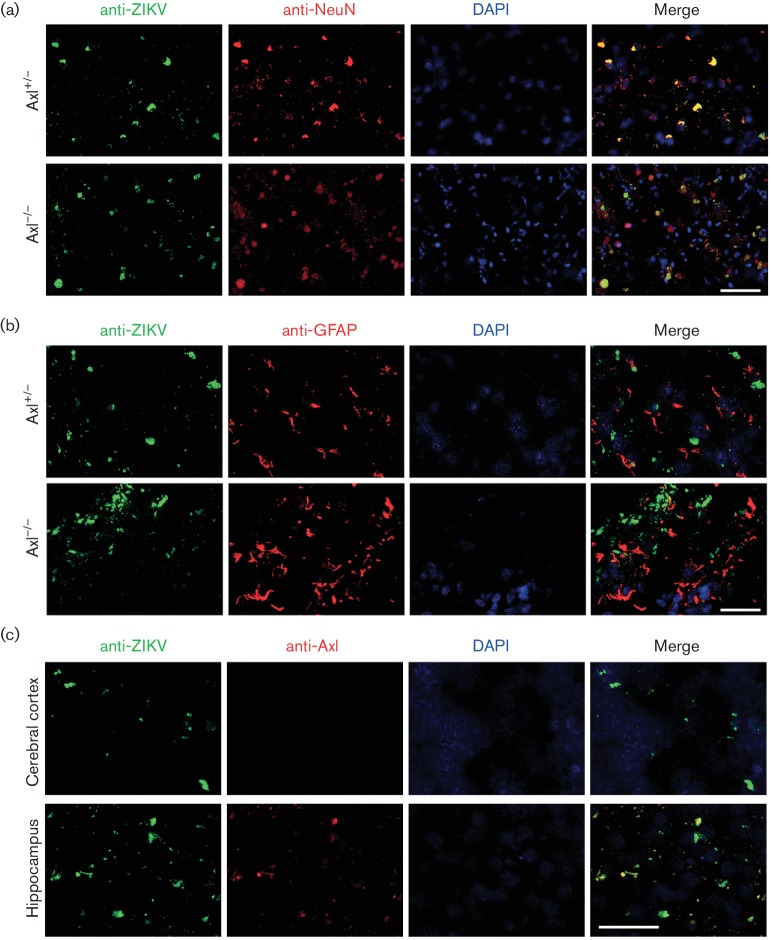
Co-immunofluorescence of ZIKV antigens and cellular markers in the brain. Axl-deficient (Axl^−/−^) newborn mice and their littermates (Axl^+/−^) were intracerebrally injected with 100 pfu ZIKV and the brains were harvested at 10 days p.i. Anti-ZIKV was co-immunofluorescently stained with an anti-NeuN antibody as a neuron marker (a) and an anti-GFAP antibody as astrocyte and ependymal cell markers (b). Anti-ZIKV was also co-immunofluorescently stained with the anti-Axl antibody, an indicator of the Axl expression level, in the brains from Axl^+/−^ mice (c). Scale bar: 50 µm.

To further study the susceptible cell types for ZIKV infection in the brain, ZIKV antigens were co-immunostained with the cellular markers of neurons or gliocytes on the brain cyrosections of Axl^−/−^ mice and their littermates. Physiologically, Axl^−/−^ mice and their littermates displayed a similar distribution pattern of NeuN, the marker of the neuron [22] as well as glial fibrillary acidic protein (GFAP), the marker of the astrocyte [23] and ependymal cell [24] (Fig. S4). In all mice, ZIKV antigens were co-localized well with NeuN (Fig. 4a), rather than with GFAP (Fig. 4b), indicating that with or without the presence of Axl, the neuron was consistently the primary target cell type for ZIKV infection. In further experiments, co-immunostaining of ZIKV and Axl was performed in brain cyrosections of Axl^+/−^ mice. Although most of the ZIKV-infected cells expressed Axl in the hippocampus (Fig. 4c, lower panels), the cells infected by ZIKV were negative for Axl in the cerebral cortex (Fig. 4c, upper panels), showing that Axl was not an indispensable factor for ZIKV infection.

## Discussion

In this study, by using Axl-deficient suckling mice and their heterozygous littermate controls, we showed that all these mice were able to support the replication of ZIKV and displayed clinical manifestations via intracerebral injection. No difference was found between Axl-deficient mice and their littermates, either in terms of the viral load, clinical manifestations, viral distribution or survival rate. Our results, therefore, provided the first infection outcomes and validated *in vivo* evidence arguing that Axl was not an indispensable factor for ZIKV infection.

Although many studies proclaimed that Axl is a receptor for ZIKV entry *in vitro*, there are indeed a few works alleging the opposite results. Miner *et al.* found that the development of conjunctivitis, panuveitis and infection of the cornea were independent of Axl in ZIKV-infected mice [20]. Govero *et al.* demonstrated high-level ZIKV infection in testis and epididymis in Axl-deficient mice [10]. In a recent study, Wells *et al.* found that the genetic ablation of Axl did not protect human neural progenitor cells from ZIKV Infection [25]. All of these results suggest that Axl may not be required for ZIKV infection in mice. Here, by characterizing in detail the infection of ZIKV in the presence or absence of Axl, we provide *in vivo* data showing that Axl has no essential role in ZIKV infection in mice. Very recently, Li and Hastings also reported that ZIKV replicated to a similar level in the brains of Axl wild-type and Axl knockout mice [26, 27]. Comprehensively, the results further support the above-mentioned view: Axl is not an indispensable factor for ZIKV infection in mice.

It is notable that our results do not necessarily mean that Axl is not a receptor for ZIKV entry; it just implies that Axl does not contribute crucially during ZIKV infection *in vivo*. As the expression, distribution and interaction of cellular surface proteins in immortal cells are often different from that in primary cells *in vivo*, it is not surprising that some viral receptors identified *in vitro* have little contribution to viral infection *in vivo*. Therefore, before the viral receptors identified *in vitro* are designed as the target for an antiviral screen, it is necessary to evaluate their *in vivo* role, at least in small animals. Moreover, the variety and complexity of receptors have been reported for many viruses. For example, DENV, one of the best-studied flavivirus, is well known for its ability to use more than one receptor for viral entry [28] including heparan sulfate [29], DC-SIGN [30], β_3_-integrin [31]as well as Axl [14]. Meanwhile, the secretion of DENV also requires a KDEL receptor for the traffic from endoplasmic reticulum to Golgi apparatus [32]. The involvement of more than one receptor is possible to occur for ZIKV, which would also compromise the impact of Axl on ZIKV infection in mice. Thus, in the future, considerably more work is required to elucidate the molecular mechanism for ZIKV infection.

## Methods

### Virus and cells

ZIKV (Asian lineage, CAS-ZK01 strain) was isolated from a patient with Zika fever and kindly provided by Dr George F. Gao (Institute of Microbiology, Chinese Academy of Sciences, Beijing, PR China). C6/36 cells (Aedes albopictus cells) were maintained at 28 °C in RPMI 1640 (Gibco, USA) supplemented with 10 % fetal bovine serum (FBS, PAN, Germany). Vero cells (African green monkey kidney cell) were maintained in minimum essential medium (MEM, Gibco, USA) supplemented with 5 % FBS at 37 °C.

ZIKV was propagated in C6/36 cells in RPMI 1640 supplemented with 2 % FBS. And the virus in cultural supernatant was collected at the fifth, sixth and seventh day p.i. The viral titres were determined by a plaque assay on Vero cell monolayers under a fresh MEM overlay containing 1.2 % methylcellulose and 2 % FBS (MEM overlay) (Fig. S5). Viral stocks were stored at −80 °C until use.

For the plaque assay, the virus stock was serially diluted and incubated with Vero cell monolayers for 2 h with moderate shakings every 30 min. Afterwards, the virus was removed and the MEM overlay was added onto the Vero monolayer, which followed by 7 days of continuous incubation at 37 °C. After the MEM overlay was removed a final crystal violet staining was then utilized to visualize plaques formed by ZIKV infection.

### Mice

Mice deficient in Axl (F0 Axl^−/−^) were kindly provided by Professor Dan-Shu Han (Institute of Basic Medical Sciences, Chinese Academy of Medical Sciences, Peking Union Medical College, Beijing, PR China). The mice were bred and maintained under a specific pathogen-free animal facility at Capital Medical University. Male F0 mice (Axl^−/−^) was mated with female SJL mice (Axl^+/+^) to produce F1 mice (Axl^+/−^). Male F0 mice (Axl^−/−^) were then mated with female F1 mice (Axl^+/−^) to produce F2 mice (Axl^−/−^ for experiments and Axl^+/−^ as littermate controls). The average age of F2 suckling mice used in this study was 1.5 days.

### Genotyping PCR

Genotyping PCR was performed using a Mouse Tissue Direct PCR Kit (KG205, Tiangen) and a set of three primers (Wt: 5′-GCCGAGGTATAGTCTGTCACAG-3′, Mut:5′-TT TGCCAAGTTCTAATTCCATC-3′, and WtMut: 5′- AGAAGGGGTTAGATGAGGAC-3′). The genotyping experiments were carried out according to the manufacturer's instruction with using a few murine tail tissues. The size of PCR products are 350 bp for Axl^+/+^ mice, 350 and 200 bp for Axl^+/−^ mice and 200 bp for Axl^−/−^ mice.

### Mouse experiments

For ZIKV infection, newborn mice were challenged with 100 pfu ZIKV in 20 µl PBS through intracerebral injection. Mice administered with 20 µl PBS served as mock controls. Survival indexes, including body weight, disease manifestation and survival rates of mice were recorded each day in the following 30 days or till death. Organs and serum of the infected mice were collected at the tenth day p.i. for determination of virus load and histological examination.

### ZIKV mRNA quantification

The infected and mock control mice were euthanized by cervical dislocation and the major organs were harvested and homogenized in Trizol (Transgen, China, ET101-01). RNA was isolated from tissue lysates according to the manufacturer’s protocol. The extracted RNA was dissolved in RNase free water and its purity and concentration were determined by NanoDrop 2000C (Thermo Scientific, USA) three times and represented as a mean value. A pair of primers (forward: 5′-TTGGGTTGTGTACGGAACCTG-3′, reverse: 5′-GTGCTTTGTGTATTCTCTTGA-3′) were designed to detect ZIKV genomic RNA. Real-time qPCR analysis was performed with Quant One Step qRT-PCR (SYBR Green I) Kit (FP303-01, Tiangen) on 7500 Real Time PCR System (Applied Biosystems, USA) according to the manufacturer’s instruction. ZIKV genome RNA (MR766) transcripted *in vitro*, kindly provided by Professor Ai-Hua Zheng from CAS, was quantified and used as a standard template to establish the standard curve. Quantification of the copies of ZIKV mRNA was determined by the standard curve method and expressed as the copy number per µg total RNA (for organs) or ml (for sera).

### Immunofluorescent staining

The whole brains were embedded in OCT (Jung, Leica) immediately after excision and processed into 5 µm thick frozen sections. The tissue slices were air-dried and then fixed in ice cold acetone. Tissue slices were permeabilized in 0.5 % Triton-X100 solution for 10 min at room temperature and blocked in 5 % BSA for 2 h at 4 °C. The tissue slices were incubated with anti-ZIKV mouse sera (1 : 200), anti-NeuN antibody (ab104225, abcam, 1 : 500), anti-GFAP antibody (ab7260, abcam, 1 : 500) and/or anti-Axl antibody (AF854, Novus Biologicals, 1 : 500) for 3 h at 37 °C or overnight at 4 °C. Goat anti-mouse IgG Alexa Fluor 488 (A-11029, Invitrogen, 1 : 500) and Goat anti-rabbit IgG Alexa Fluor 594 (R37117, Invitrogen, 1 : 500) were used as secondary antibodies for 1 h at 37 °C. DAPI was used to display cell nuclei. All images were captured with a laser scanning confocal microscopy (Leica TCS SP5).

### Hematoxylin and eosin staining

The ZIKV-challenged mice were euthanized by cervical dislocation at 10 days p.i. and the major organs were harvested. The organs were divided into two parts. One was immediately fixed in modified Davidson’s fluid solution (30 ml of 40 % formaldehyde, 15 ml of ethanol, 5 ml of glacial acetic acid and 50 ml of distilled water) overnight. Then the organs were embedded in paraffin, sectioned at 5 µm in thickness and subjected to a standard hematoxylin and eosin staining. The others were embedded in OCT (Jung, Leica) immediately and then subjected to 5 µm thick frozen sections for detection of ZIKV antigens by immunehistochemical staining.

### Statistical analysis

Statistical analysis was performed with SPSS 19.0. Two-way ANOVA and log rank test were used to compare the body weight changes and survival rates of the two groups, respectively. The quantitative data of the two groups were compared using Student's *t*-test. The survival rates of the two groups were compared with calibrated Chi square test. Differences among the groups were considered to be significant at *P*<0.05.

## Supplementary Data

1038 Supplementary File 1
