# Supplementary material for: Axl is not an indispensable factor for Zika virus infection in mice
Source: J Gen Virol. 2017 Aug 8;98(8):2061–8. doi: 10.1099/jgv.0.000886 (PMC5656784; doi:10.1099/jgv.0.000886)
Supplement: Supplementary File 1 [file jgv-98-2061-s001.pdf]

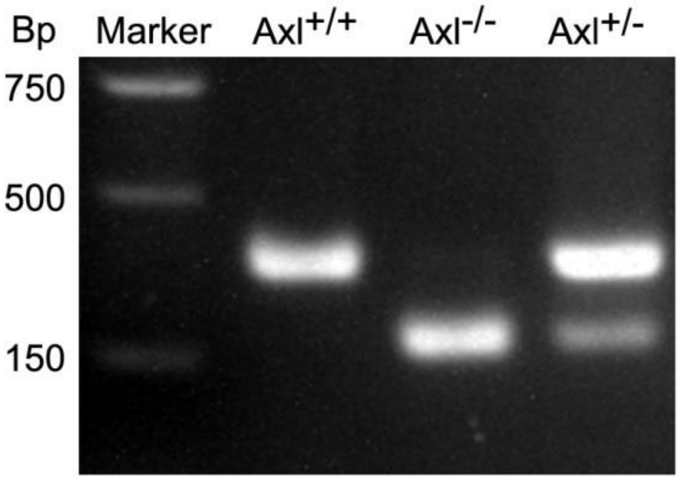

Supplementary Figure 1. Representative picture of genotyping PCR results. The sizes of PCR products were 350 bp for $Axl^{+/+}$, 200bp for $Axl^{-/-}$ and 350 bp and 200 bp for $Axl^{+/-}$. Related to Figure 1 in the main text.

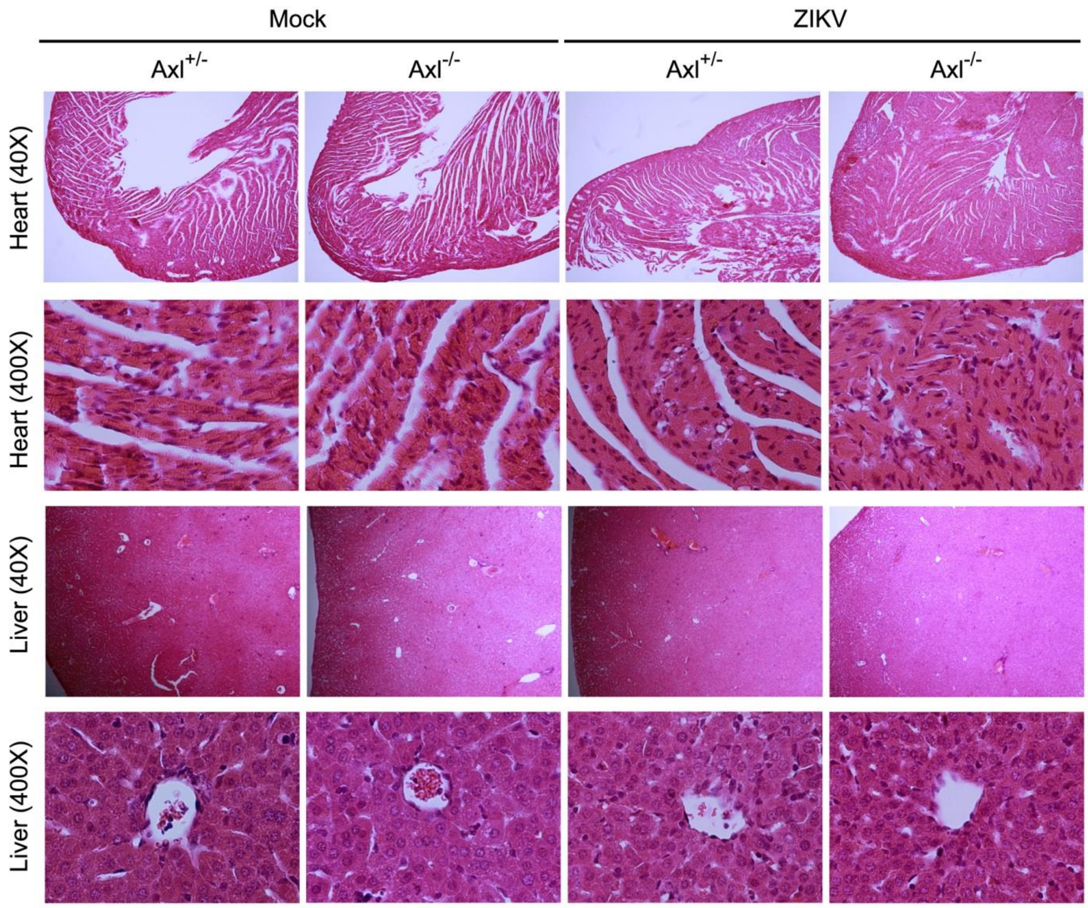

Supplementary Figure 2. H&E staining of the heart and liver from ZIKV-infected mice. Axl deficient ($Axl^{-/-}$) newborn mice and their littermates ($Axl^{+/-}$) were intracerebrally injected with 100 pfu ZIKV or same volume of PBS (mock). The heart and liver were harvested at 10 dpi and processed for H&E staining. Magnification: 40X or 400X as indicated. Related to Figure 2 in the main text.

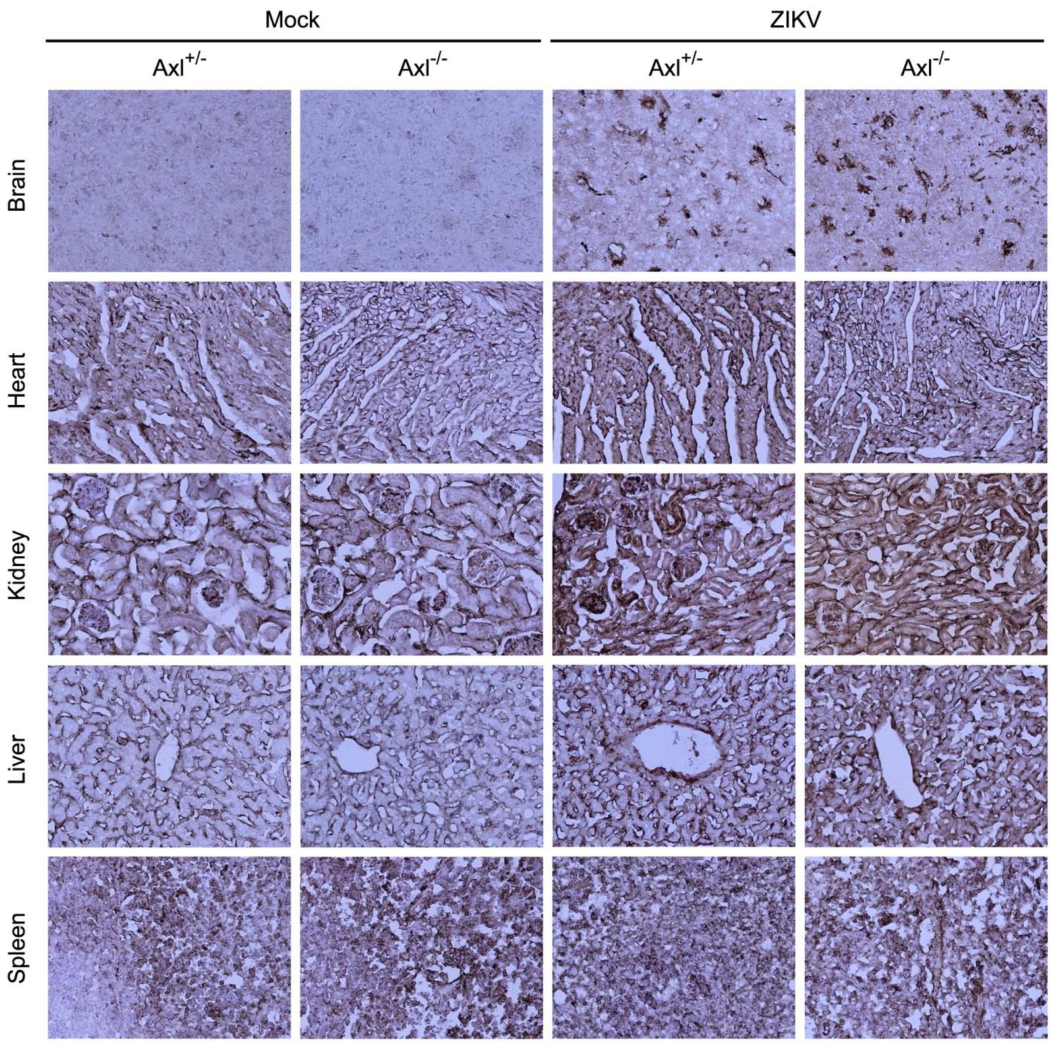

Supplementary Figure 3. Immunohistochemical staining of ZIKV antigen in the representative organs. Axl deficient ($Axl^{-/-}$) newborn mice and their littermates ($Axl^{+/-}$) were intracerebrally injected with 100 pfu ZIKV or the same volume of PBS (mock). The brain, heart, kidney, liver and spleen were harvested at 10 dpi and processed for immunohistochemical staining of ZIKV antigen as indicated. Magnification: 200X. Related to Figure 3 in the main text.

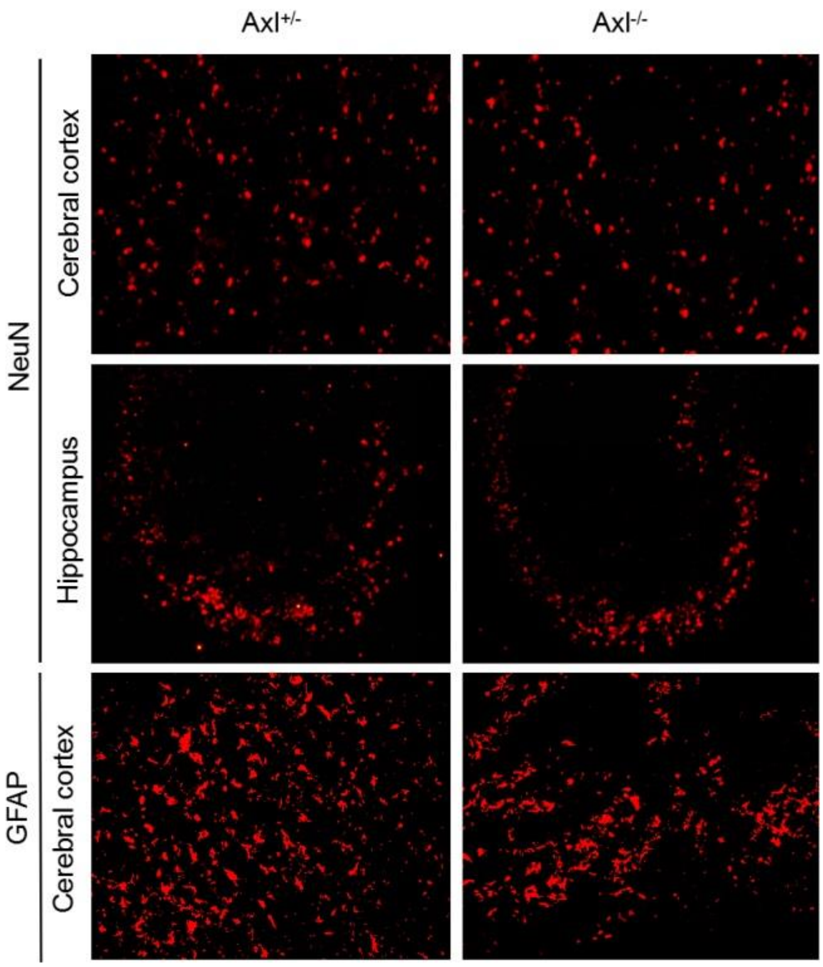

Supplementary Figure 4. Physiological distribution pattern of NeuN and GFAP in brains from Axl deficient mice and their littermates. The brain cryosections from 10 days old uninfected Axl deficient ($Axl^{-/-}$) newborn mice and their littermates ($Axl^{+/-}$) were subjected to immunofluorescent staining of NeuN, the marker of neuron, and GFAP, the marker of astrocyte and ependymal cell. Magnification: 100X. Related to Figure 4 in the main text.

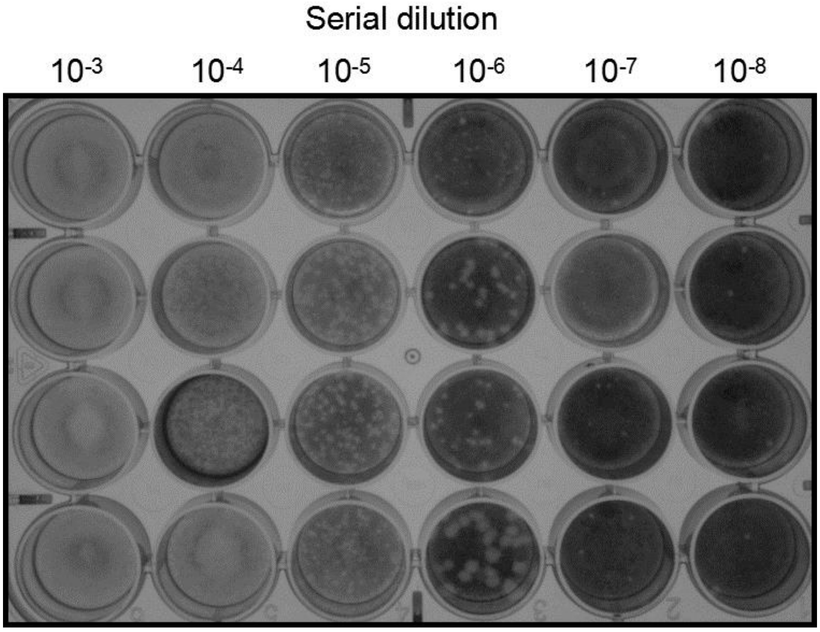

Supplementary Figure 5. Plaque assay to determine the viral titer of ZIKV. ZIKV used for animal injection was serially diluted and incubated on Vero cell monolayer. A final crystal violet staining was then utilized to visualize the plaques formed by ZIKV infection.
